# STAT3 in the dorsal raphe gates behavioural reactivity and regulates gene networks associated with psychopathology

**DOI:** 10.1038/s41380-020-00904-2

**Published:** 2020-10-12

**Authors:** Sonali N. Reisinger, Spyros Sideromenos, Orsolya Horvath, Sophia Derdak, Ana Cicvaric, Francisco J. Monje, Martin Bilban, Martin Häring, Micaela Glat, Daniela D. Pollak

**Affiliations:** 1 Department of Neurophysiology and Neuropharmacology, Center for Physiology and Pharmacology, Medical University of Vienna, Schwarzspanierstraße 17, 1090 Vienna, Austria; 2 Core Facilities Genomics, Medical University of Vienna, Lazarettgasse 14, 1090 Vienna, Austria; 3 Department of Laboratory Medicine, Medical University of Vienna, Währinger Gürtel 18-20, 1090 Vienna, Austria; 4 Department of Molecular Neurosciences, Center for Brain Research, Medical University of Vienna, Spitalgasse 4, 1090 Vienna, Austria

**Keywords:** Neuroscience, Depression

## Abstract

The signal transducer and activator of transcription 3 (STAT3) signalling pathway is activated through phosphorylation by Janus kinases in response to a diverse set of immunogenic and non-immunogenic triggers. Several distinct lines of evidence propose an intricate involvement of STAT3 in neural function relevant to behaviour in health and disease. However, in part due to the pleiotropic effects resulting from its DNA binding activity and the consequent regulation of expression of a variety of genes with context-dependent cellular consequences, the precise nature of STAT3 involvement in the neural mechanisms underlying psychopathology remains incompletely understood. Here, we focused on the midbrain serotonergic system, a central hub for the regulation of emotions, to examine the relevance of STAT3 signalling for emotional behaviour in mice by selectively knocking down raphe STAT3 expression using germline genetic (STAT3 KO) and viral-mediated approaches. Mice lacking serotonergic STAT3 presented with reduced negative behavioural reactivity and a blunted response to the sensitising effects of amphetamine, alongside alterations in midbrain neuronal firing activity of serotonergic neurons and transcriptional control of gene networks relevant for neuropsychiatric disorders. Viral knockdown of dorsal raphe (DR) STAT3 phenocopied the behavioural alterations of STAT3 KO mice, excluding a developmentally determined effect and suggesting that disruption of STAT3 signalling in the DR of adult mice is sufficient for the manifestation of behavioural traits relevant to psychopathology. Collectively, these results suggest DR STAT3 as a molecular gate for the control of behavioural reactivity, constituting a mechanistic link between the upstream activators of STAT3, serotonergic neurotransmission and psychopathology.

## Introduction

Globally, 350 million people suffer from major depressive disorder (MDD), which is estimated to constitute the number one cause of disease burden by 2030. However, MDD is not the only severe mental illness presenting with an epidemic propagation. 60 million people worldwide are confronted with a diagnosis of bipolar depression (BP) and patients suffering from schizophrenia (SCZ) are estimated at 21 million (available from: http://www.who.com). Despite half a century of intensive research, our insight into the pathophysiological principles underlying these excruciating conditions remains incomplete, thus limiting the development of urgently needed novel diagnostic and therapeutic strategies. Efforts originating from the Research Domain Criteria concept, launched by the National Institute of Mental Health in 2008 [1], suggest a research program focused on the investigation of individual constructs of behaviour and brain function, rather than disease models of psychiatric disorders. Others have suggested a bottom-up strategy, centred on the identification and exploration of individual aetiological factors, to be more effective [2].

Here we used a combinatorial approach to examine the involvement of a specific molecular element, Signal transducer and activator of transcription 3 (STAT3), for behavioural phenotypes with relevance across traditional psychiatric disease entities and to interrogate the underlying neural principles. Activation of the transcription factor STAT3 is consequent to an intracellular molecular cascade involving activation of Janus kinase (JAK) in response to a diverse set of immunogenic and non-immunogenic stimuli, ultimately culminating in the phosphorylation and dimerisation of STAT3. Upon phosphorylation and dimerisation, STAT3 translocates to the nucleus to regulate gene expression directly, or by forming complexes with other transcription factors [3, 4].

The focus on STAT3 and its dependent molecular pathways originated from a set of observations which collectively propose an intricate involvement in neural function relevant to behaviour in health and disease: (i) STAT3 is a major orchestrator of cellular immune processes in response to cytokine activation, which has been highly implicated in psychopathology within the framework of the “immune hypothesis” [5–10]; (ii) besides cytokines, STAT3 activation can reflect the action of many different upstream regulators with relevance for neural function, including growth factors, hormones and endocannabinoids [11–19]; (iii) STAT3 importantly influences synaptic plasticity-related events [20–22] and can act to control gene expression through epigenetic processes [23–25], hereby potentially impacting on neural mechanisms relevant for psychopathology independently of its genomic activities or immune stimulation; (iv). Several indications for an interrelationship between STAT3 and the serotonergic neurotransmission system, highly implicated in the aetiology of psychiatric disorders, have also recently emerged [26, 27]. Indeed, a role for STAT3 in psychopathology, respectively in relevant animal models thereof, has been reported by our own group and others [26–32], overall suggesting STAT3 signalling as a regulatory lever for the expression of negative emotional behaviour and the response to aversive conditions. Due to this converging evidence, we hypothesised an important role for STAT3 signalling, specifically within the serotonergic system of the brain, in the regulation of behavioural traits relevant to mental illness. We set out to experimentally address this question using a serotonergic cell-specific STAT3 knockout mouse (*Sert*^Cre/+^; *Stat3*^fl/fl^). A thorough behavioural characterisation of this mouse model, together with in vivo electrophysiological recordings and transcriptomic analysis of the dorsal raphe (DR), where most serotonergic cell bodies are located [33], was complemented by the corresponding behavioural evaluation of the consequences following viral-mediated, short-term knockdown of STAT3 in the adult DR. Collectively, the findings obtained provide convincing evidence that STAT3 works as a molecular switch in the DR governing the expression of gene networks central to psychopathology, with consequences for neuronal function and reflected in a reduced negative behavioural reactivity to aversive conditions and a parallel diminished response to the sensitising effects of amphetamine.

## Materials and methods

Please see the supplementary materials and methods for a comprehensive description of all procedures. A list of sample sizes employed in each experiment can be found in Supplementary Table 1. Sample sizes were determined according to own and other´s published results of comparable studies [34–37]. During all experiments prior to statistical analysis, animals´ identities were numerically encoded, allowing the experimenter to be blinded to the genotype or experimental condition of each subject.

### Animals

Conditional STAT3 knockout mice *Sert*^Cre/+^; *Stat3*^fl/fl^ (“KO”) and littermate *Sert*^Cre/+^; *Stat3*^fl/+^ (“control”) were bred from founder pairs of B6.129S1-Stat3 < tm1Xyfu > /J (JAX stock #016923, Jackson Laboratories, Bar Harbour, ME, USA) [38] and B6.129(Cg)-Slc6a4 < tm1(cre)Xz > /Cnrm (European Mouse Mutant Archive, Monterotondo Scalo, Rome, ITA) [39]. *Sert*^+/+^; *Stat3*^fl/fl^ were employed for viral knockdown experiments. Mice were kept on a 12/12 hour light/dark cycle in a temperature-controlled colony room (22 ± 1 °C) and provided with food and water *ad libitum*. Single-housed male and female littermate mice (8–16 weeks at experiment onset) were used for all experiments. All animal experiments were conducted adhering to the European Communities Council Directive of September 22, 2010 (2010/63/EU) after approval by the national authorities (Austrian Federal Ministry of Education, Science and Research, no. BMBWF-66.009/0175-V/3b/2019).

### In vivo electrophysiology of DR 5-HT neurons

In vivo recordings of DR 5-hydroxytryptamine (5-HT, serotonin) neurons were essentially conducted as previously reported [36].

An Axoclamp-2B amplifier was used to obtain extracellular recordings and analogue-to-digital data conversion was done using the Digidata-1440 interface. Data processing and analysis were performed using the software pClamp-10 and Clampfit, respectively (all apparatus and software: Axon Instruments, Union City, USA).

### Behaviour

Preliminary analysis of STAT3 KO behaviours revealed no genotype × sex interactions, therefore mice of both sexes were collapsed into a single group of each genotype, and mixed-sex groups were used in all other experiments.

#### Sucrose preference test (SPT)

The SPT followed an established protocol [36] and relative sucrose preference (% of total liquid consumption) was calculated and used as indicator of hedonic behaviour [40].

#### Novelty-suppressed feeding (NSF)

The NSF was conducted according to a published procedure [36] and the latency to the first bite (s) from a pellet placed in a brightly-lit arena was used as relevant parameter [41].

#### Forced swim test (FST)

The FST was carried out using automated movement tracking software (VideoTrack v3, Viewpoint, Champagne au Mont d’Or, France) as reported earlier and the relative immobility (%) was calculated [36].

#### Open field test (OFT)

The OFT was performed to measure spontaneous locomotor activity (total distance travelled, m) [36] in arenas fitted with laser beams for automatic movement tracking and analysis (Activity Monitor v5, MedAssociates, Fairfax, USA).

#### Light-dark box (LDB)

The LDB was used to evaluate anxiety-like behaviour (time spent in the aversive light zone, %) [36, 42] employing an automated tracking and analysis system (Activity Monitor v5, MedAssociates).

#### Elevated plus maze (EPM)

Anxiety-like behaviour in the EPM (time spent in open arms, %) was determined as previously described [36, 43] using Videotrack (Viewpoint).

#### Rotarod (RR)

Motor coordination was tested using an automatic RR system for rodents (MedAssociates) [36]. Average latency to fall (s) was determined over 3 trials.

#### Amphetamine sensitisation

Locomotor sensitisation (% change in distance travelled from day 1) was evaluated in the open field (apparatus as above) for 30 minutes after i.p. injection of *d-*amphetamine (2 mg/kg free base, 10 ml/kg volume) (protocol adapted from [44]).

#### Conditioned place preference (CPP)

CPP was performed using a biased experimental paradigm [45] employing *d*-amphetamine (5 mg/kg free base, 10 ml/kg volume i.p.) or saline injections (0.9%, 10 ml/kg volume i.p.). Side preference (%) on the test day was calculated after 6 days of training.

### Drugs

*D*-amphetamine sulphate (GlaxoSmithKline, London, UK) was dissolved in physiological saline (0.9%) for administration during amphetamine sensitisation and CPP. To obtain the indicated free base concentrations, a conversion factor of 1.36 was applied to calculate the amount of *d*-amphetamine sulphate needed.

### Immunohistology and quantification of fluorescence

Mice were anaesthetised (ketamine 100 mg/kg; xylazine: 40 mg/kg; i.p., both 10 ml/ kg) and transcardially perfused using 4% paraformaldehyde in PBS. 30 µm sections of the DR (Bregma −4.0 mm to −5.2 mm [46]) were collected on a cryostat and used for double 5-HT/STAT3, Iba1/STAT3, GFAP/STAT3, STAT3 and NeuN stainings (primary antibodies: goat anti-5-HT, Abcam, ab66047, 1:200; rabbit anti-STAT3, Santa Cruz Biotechnology, sc-7179, 1:100; rabbit anti-Iba1, Fujifilm Wako Pure Chemical Corporation, 019-19741, 1:1000; rabbit anti-GFAP, G4546, Sigma-Aldrich, 1:500; mouse anti-STAT3, Abcam, ab119352, 1:100; mouse anti-NeuN, Chemicon/ Sigma-Aldrich, MAB377, 1:500; secondary antibodies: donkey anti-goat, Alexa 488, Invitrogen, A11055, 1:200; donkey anti-rabbit, Alexa 594, Invitrogen, A21207, 1:500; goat anti-mouse, CF 594, Merck, SAB4600402, 1:1000). An overview of the primary antibodies employed can be found in Supplementary Table 2.

Images were obtained using a Nikon A1 laser scanning microscope and the software NIS-Elements AR (version 5.02.01, Nikon Instruments Inc., Tokyo, JP) using 20X, 60X or 100X objectives. Quantification of STAT3 immunoreactivity within the ROI or within specific cell types was performed using the software ImageJ [47, 48]. Briefly, quantification of 5-HT/STAT3, Iba1/STAT3 and GFAP/STAT3 experiments was conducted in areas expressing 5-HT, Iba1 or GFAP, i.e., serotonergic cells, microglia or astrocytes, respectively. Selection masks were created via thresholding single-channel images of these immunostainings and were subsequently applied to the corresponding single-channel images of STAT3 immunoreactivity as “ROIs”. In these images STAT3 signal intensities in each of the relevant cell types was quantified and compared between groups. For the quantification of STAT3 expression in the DR after viral delivery of Cre recombinase or GFP respectively, STAT3 signal intensity was quantified using ImageJ in a rectangular ROI corresponding to the DR (ca. 700 × 800 µm^2^; ventral to cerebral aqueduct at midline) and obtained values were compared between groups.

### Brain extraction

Mice were killed by cervical dislocation, whole brains were extracted, rapidly embedded in O.C.T. medium (Sakura Finetek, Staufen im Breisgau, GER) and stored at −80 °C. The DR was collected from four 300 µm-thick coronal sections (Bregma −4.0 mm to Bregma −5.2 mm [46]) by dissecting a square area (1.5 × 1.5 mm^2^) directly below the aqueduct [46]. Tissue was stored in RNALater (ThermoFisherScientific, Vienna, AUT) at −20 °C.

### RNA isolation

RNA extraction was performed using miRNEasy Mini Kit (Qiagen, Venlo, NL) by following the manufacturer’s protocol precisely (“Quick start guide”). RNA was eluted from the column filter in the final step using 40 µl of RNAse-free water.

### RNA-Seq and bioinformatic analysis

Samples were quality-checked using a Bioanalyzer (Agilent Technologies, Santa Clara, USA) and showed RNA Integrity Numbers above 7.0. Library preparation and sequencing was performed as previously described [49]. Data were analysed on the Illumina Basespace platform (Illumina, San Diego, USA) using the RNA-Seq alignment app and followed up using STAR aligner [50] and DESeq2 [51]. Raw *p* values were corrected by applying the Benjamini-Hochberg method and a false discovery rate of 5% to produce *q* values.

The full dataset is available online in the NCBI GEO (gene expression omnibus) functional genomics data repository under the accession number GSE146101.

### Quantitative Real Time-PCR

450 ng of DR RNA were used for cDNA synthesis using the DyNamo cDNA Synthesis Kit (ThermoFisherScientific) by following the manufacturer’s instructions. STAT3 expression relative to β-actin expression were then determined using qRT-PCR using the ΔΔC(t) method [52]. Primer sequences are available in Supplementary Table 2.

### Viral knockdown STAT3 in midbrain DR

Male and female *Stat3*^fl/fl^ mice were deeply anaesthetised with isoflurane. 0.5 µl of AAV-Cre (pAAV.CMV.HI.eGFP-Cre.WPRE.SV40, Addgene viral prep #105545-AAV5) or AAV-GFP (pAAV.CMV.PI.EGFP.WPRE.bGH; Addgene viral prep #105530-AAV5; produced by James M. Wilson; titre: 10^13^ genome copies/ ml) were infused over 8 min at each of the following coordinates (adjusted for each mouse using a conversion factor *f* as outlined in [53]): (a) *x* = 0.0, *y* = −4.5, z = −2.9; (b) *x* = 0.0, *y* = −4.5, *z* = −2.7.

Upon completion of behavioural testing mice were transcardially perfused and 30 µm DR sections were examined for the overall spread of GFP+ cells.

### Statistical analysis

GraphPad Prism 7.0 (GraphPad Software Inc., San Diego, USA) was used for all statistical analyses and preparation of graphs. Statistical outliers falling outside the interval mean ± 2 SD were pre-excluded from analyses to avoid skewing the data, as this interval represents >95% of values around the mean of a normally distributed dataset. Two-tailed, two-sample Student’s *t* test was applied wherever two groups were being compared (with Welch correction where *F*-test indicated unequal variances) and in the case of a repeated measures design, repeated-measure ANOVA was applied.

## Results

### Serotonergic STAT3 knockout alters DR neuronal firing rate

STAT3 conditional knockout mice (*Sert*^Cre/+^; *Stat3*^fl/fl^; hereinafter “KO”) bearing a STAT3 deletion in all SERT-expressing cells, i.e., predominantly serotonergic neurons of the midbrain DR [33, 54], were used here to examine the effects of STAT3 disruption on aspects of physiology and behaviour relevant to psychopathology.

**Fig. 1:**
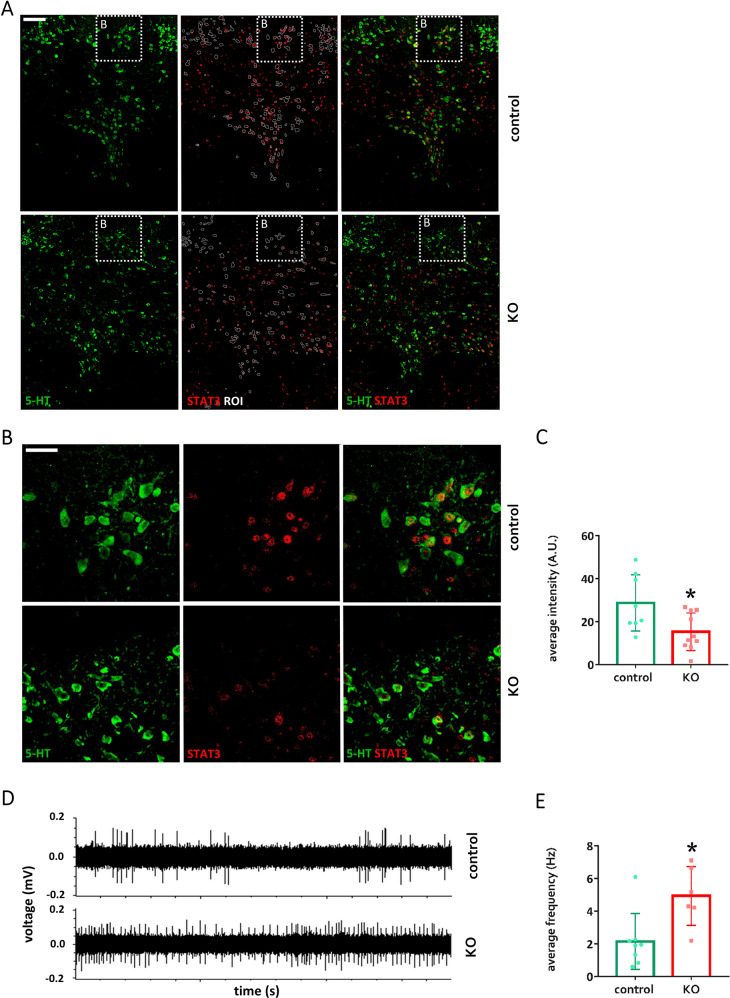
Loss of serotonergic STAT3 alters 5-HT neuronal excitability in vivo. **A** STAT3 immunoreactivity was evaluated within 5-HT-positive cells (ROI: white outlines shown in STAT3 channel) in images of the DR area (representative 4 × 5 stitched 60X images; scale bar: 100 µm), illustrating a visible reduction of STAT3 signal within the specified ROI (i.e., serotonergic cells) in KO animals. White dashed boxes mark the detailed area depicted in (**B**). **B** Corresponding single panel images of 5-HT and STAT3 immunoreactivity and a merged image in control and KO mice (60X, scale bar: 50 µm). **C** Quantification of STAT3 immunoreactivity within the ROI confirmed the reduction of STAT3 expression in KO serotonergic neurons of the DR. **D**, **E** STAT3 KO mice displayed significantly increased serotonergic firing frequencies compared to controls. 5-HT: 5-hydroxytryptamine; A.U.: arbitrary units; DR: dorsal raphe; KO: knockout; ROI: region of interest; STAT3: signal transducer and activator of transcription 3. All data are presented as mean ± SD. *p < 0.05.

STAT3 expression in DR 5-HT neurons of KO and control mice was evaluated by specific immunoreactivity in brain sections. STAT3 fluorescence intensity was quantified within 5-HT-labelled cells (mask defined as the “ROI”; Fig. 1A) and revealed a significant reduction of STAT3 within serotonergic neurons of the KO DR (Fig. 1B, C; t_16_ = 2.607; *p* = 0.019; *n* = 8–10/group). However, STAT3 immunoreactivity in microglia (Supplementary Fig. 1A, B; *t*_16_ = 0.751; *p* = 0.4636; *n* = 9/group) and astrocytes (Supplementary Fig. 1C, D; *t*_16_ = 0.101; *p* = 0.921; *n* = 9/group) was generally low and showed no differences between groups.

In vivo electrophysiological recordings of extracellular action potentials in this region revealed significantly higher average firing rates of serotonergic neurons in KO compared to controls (Fig. 1D, E; *t*_12_ = 2.960; *p* = 0.012; *n* = 6–8/group), suggesting an impact of STAT3 deletion on intrinsic excitability features of 5-HT neurons.

### Absence of serotonergic STAT3 results in a more active coping style and reduced negative emotional displays under stressful conditions

**Fig. 2:**
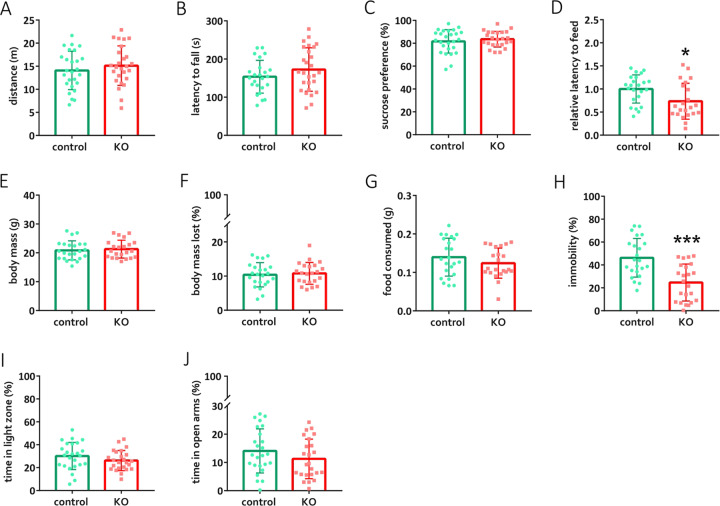
Loss of serotonergic STAT3 leads to differential behavioural reactivity in stressful behavioural paradigms. Motor function was not impacted by knockout of STAT3 in serotonergic cells, as (**A**) locomotor activity in the OFT and (**B**) motor coordination in the RR were similar between groups. **C** No differences between genotypes in sucrose preference were found in the SPT. **D** Average latency to feed in an adverse environment was reduced in KO animals relative to controls in the NSF. No effect of serotonergic STAT3 knockout on (**E**) body mass, (**F**) body mass lost during the food restriction period preceding the NSF, or (**G**) food consumption immediately after the NSF test. **H** KO animals showed markedly reduced passive immobility behaviour in the FST in comparison to controls. Avoidance of the adverse compartments in (**I**) LDB and (**J**) EPM were unchanged by serotonergic STAT3 knockout. EPM: elevated plus maze; FST: forced swim test; KO: knockout; LDB: light-dark box; NSF: novelty-suppressed feeding; OFT: open field test; RR: Rotarod; SPT: sucrose preference test; STAT3: signal transducer and activator of transcription 3. All data are presented as mean ± SD. *p < 0.05; ***p < 0.001.

In light of the reported association between STAT3 signalling and mental illnesses [26–29], we next subjected STAT3 KO mice to extensive behavioural testing, with a particular focus on paradigms examining emotional behaviours relevant to depression. Baseline locomotor activity in the OFT was comparable between genotypes as control and KO groups travelled similar total distances (Fig. 2A; *t*_50_ = 0.892; *p* = 0.377; *n* = 26/group). Furthermore, KO animals did not display any alterations in motor coordination in the RR (Fig. 2B; *t*_49_ = 1.317; *p* = 0.194, *n* = 24–27/group). Serotonergic STAT3 knockout did not influence hedonic behaviour in the SPT (Fig. 2C; *t*_48_ = 0.759; *p* = 0.452; *n* = 24–26/group). In contrast, STAT3 KO mice did behave differently than control animals in the tests probing emotional behaviour under stressful conditions: in the NSF, KO mice displayed shorter latencies to feed from a food pellet placed in the centre of a brightly lit arena, indicating that when the drive to eat is contrasted by the fear of an illuminated open space in a novel environment, KO animals are more ready and able to override fear in order to terminate the conflict situation (Fig. 2D; *t*_43_ = 2.557; *p* = 0.014; *n* = 22–23/group). Importantly, this behavioural effect was not driven by overt differences in metabolism or hunger between groups: Neither individual body weight (Fig. 2E; *t*_43_ = 0.454; *p* = 0.652; *n* = 22–23/group), nor relative weight loss during the 24-h food restriction period (Fig. 2F; *t*_43_ = 0.404; *p* = 0.688; *n* = 22–23/group), nor food consumption immediately post-NSF (Fig. 2G; *t*_43_ = 1.184; *p* = 0.243; *n* = 22–23/group) were impacted by serotonergic STAT3 deletion. In the FST, mice lacking serotonergic STAT3 displayed lower levels of immobility than controls (Fig. 2H; *t*_44_ = 4.430; *p* < 0.0001; *n* = 23/group), evidencing an augmented proactive coping style of the KO group when faced with the adversity of inescapable stress [55]. Anxiety-like behaviour in the LDB and the EPM was similar between groups (Fig. 2I, J; LDB: *t*_47_ = 1.324; *p* = 0.192; *n* = 24–25/group; EPM: *t*_47_ = 1.324; *p* = 0.192; *n* = 24–25/group), suggesting that STAT3 knockout in serotonergic neurons does not influence anxiety-like behaviour in a situation of free choice.

### Transcripts altered in DR of mice lacking serotonergic STAT3 encompass a multitude of genes and gene sets associated with severe mental illnesses

**Fig. 3:**
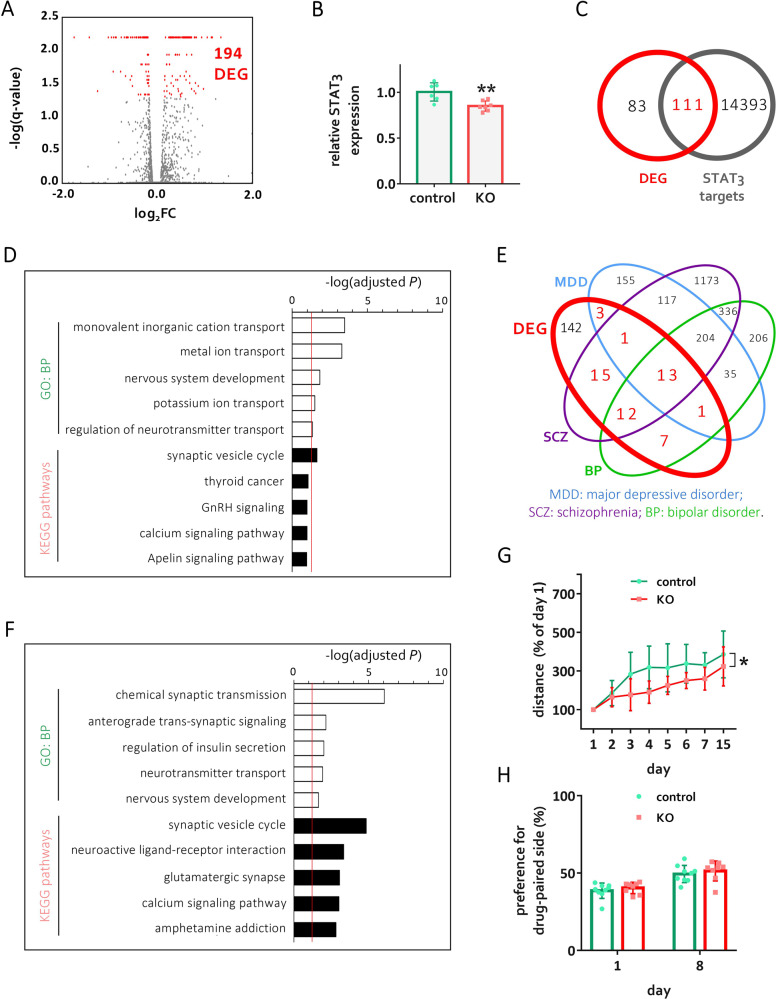
Transcriptional networks altered by serotonergic STAT3 deletion overlap with gene sets and pathways functionally implicated in psychopathology. **A** Results of RNA-Seq in DR tissue of STAT3 KO and control animals revealed 194 differentially expressed genes (DEG, red dots: *q* < 0.05). **B** qRT-PCR targeting recombined exons (18, 19, 20) of STAT3 confirms reduced expression of functional STAT3 mRNA vs. β-actin in the DR of the STAT3 KO group. **C** Of the DEG, 111 transcripts (DEG×STAT3) are reported to be direct targets of STAT3 in published ChIP-Seq experiments according to ChIP-Atlas. **D** Several gene sets relating to different aspects of synaptic transmission as well as nervous system development (GO: biological processes, white bars) and genes belonging to the synaptic vesicle KEGG pathway (black bars) were disproportionately represented among DEG×STAT3. **E** Venn analysis using lists of MDD-, SCZ- and BP- associated genes in combination with the DEG set showed 52 genes differentially expressed in KO mice which were previously associated with SMI (DEG×SMI). **F** Significantly enriched gene sets related predominantly to biological processes (white bars) and KEGG pathways (black bars) involved in neuronal excitability and cell-to-cell communication. **G** Daily *d*-amphetamine administration induced locomotor sensitisation in control mice, but this effect was significantly blunted in KO. **H** Levels of reward-associated learning in the CPP were not impacted by STAT3 loss. BP: bipolar disorder; CPP: conditioned place preference; DEG: differentially expressed genes; GO: gene ontology; KEGG: Kyoto Encyclopaedia of Genes and Genomes; KO: knockout; MDD: major depressive disorder; SCZ: schizophrenia; SMI: severe mental illnesses; STAT3: signal transducer and activator of transcription 3. All data are presented as mean ± SD. Red line in (**D**) and (**F**) denotes -log(adjusted *p* = 0.05). *p < 0.05; **p < 0.01.

We next examined molecular patterns paralleling the behavioural consequences of STAT3 deletion in the DR. mRNA sequencing was chosen as a bias-free approach to determine differences in gene expression between controls and STAT3 KO. Initial analysis revealed 194 significantly differentially expressed genes (DEG) (Fig. 3A, red dots; full results in Supplementary Table 3A). We specifically examined STAT3 mRNA expression in the same area using qRT-PCR, selectively targeting the exons deleted in STAT3 KO which contain the SH2 domain necessary for STAT3 dimerisation [38]. We confirmed significantly reduced STAT3 expression in this group (Fig. 3B; *t*_11_ = 3.556; *p* = 0.005; *n* = 6–7/group). Of the 194 DEG, we found that 111 are experimentally validated STAT3 targets (DEG×STAT3) according to ChIP-Atlas [56] (Fig. 3C). While this does not necessarily imply that dysregulation of these genes is responsible for the observed behavioural phenotype, we were most interested in the particular features and functional classifications of gene sets under direct control of STAT3. Gene list enrichment analysis using Enrichr [57, 58] revealed the 111 DEG×STAT3 to be disproportionately related to several gene ontology (GO) terms, including several GO categories associated with particular subtypes of ion transport, as well as the GO terms “neurotransmitter transport” and “nervous system development” (Fig. 3D, white bars). Looking at KEGG (Kyoto Encyclopedia of Genes and Genomes) pathways, we found that DEG×STAT3 genes were significantly enriched in genes belonging to the “synaptic vesicle cycle” pathway (Fig. 3D, black bars; both: full results in Supplementary Table 3B). We next consulted a database of publicly available disease-gene associations (DisGeNet [59]) for DEG previously associated with three severe mental illnesses (MDD, SCZ and BP; full list of genes in Supplementary Table 3C). Surprisingly, 52 (~27%) out of 194 DEG in the DR of STAT3 KO have been reported in association with MDD, SCZ and/or BP in at least one publication (DEG×SMI, Fig. 3E). We performed gene list enrichment analysis on this array of genes to identify gene sets or pathways which, by virtue of comprising genes that have previously been associated with major mental illnesses, may have a plausible likelihood of being functionally involved in the expression of the behavioural phenotype observed here. Enrichr analysis of the DEG×SMI gene set, focusing again on biological processes (GO terms, Fig. 3F, white bars) and KEGG pathways (Fig. 3F, black bars; both: full results in Supplementary Table 3D) yielded a number of significantly enriched gene sets. In particular, several of the relatively enriched gene sets again related to processes and pathways involved in neuronal signalling and synaptic function, and “nervous system development” was once more overrepresented in the DEG×SMI gene set. Intriguingly, among significantly enriched KEGG pathways, the “amphetamine addiction” pathway was also present. Since substance use disorder is highly comorbid with other forms of mental illnesses relevant to the observed behaviours [60], this association appeared particularly poignant and we hypothesised that STAT3 KO mice may by extension show altered behaviour relating to amphetamine addiction. To test this hypothesis, we performed two established behavioural tests involving amphetamine administration, each probing different aspects of the psychostimulant’s action on the brain. In the locomotor sensitisation test, the locomotor-potentiating effects of amphetamine were blunted in the KO group (Fig. 3G; STAT3 knockout – *F*_1,15_ = 5.763; *p* = 0.029; sensitisation – *F*_7,105_ = 36.310; *p* < 0.0001; STAT3 knockout x sensitisation – *F*_7,105_ = 2.652; *p* = 0.015; *n* = 8–9/group). In contrast, in the CPP both groups showed similar levels of preference for the drug-paired chamber after conditioning (Fig. 3H; STAT3 knockout – *F*_1,15_ = 0.821; *p* = 0.380; conditioning – *F*_1,15_ = 53.7; *p* < 0.0001; STAT3 knockout x conditioning – *F*_1,15_ = 0.0004; *p* = 0.9486; *n* = 8–9/group).

### Viral knockdown of STAT3 in DR of adult mice recapitulates the emotional behavioural phenotype observed in the constitutive STAT3 knockout

**Fig. 4:**
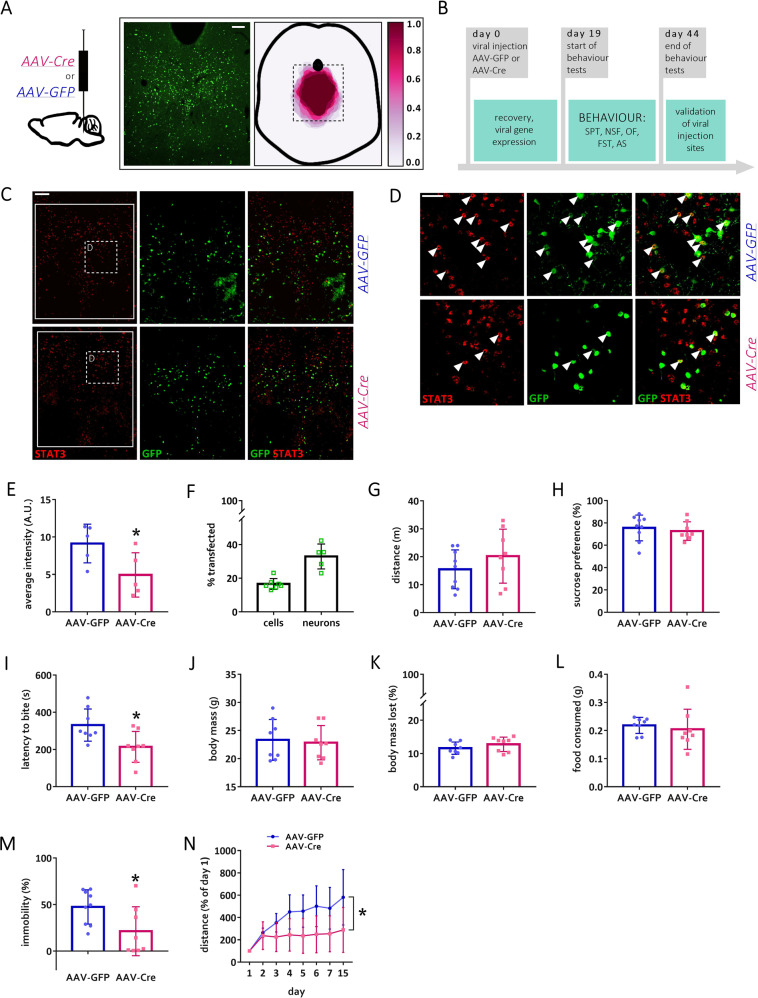
Viral-mediated knockdown of STAT3 in DR recapitulates behaviours relevant to psychopathology observed in serotonergic STAT3 KO mouse. **A** Adult *Stat3*^*fl/fl*^ mice were injected with AAV-Cre or AAV-GFP in the DR. A representative image (left panel; 10X, scale bar: 100 µm; area represented by box in right panel) and a heat map (right panel) showing the overlap of transfected areas in the cohort of AAV-Cre-injected mice are shown, with the darkest area representing the area that was targeted in all animals. **B** Timeline of experiments and procedures for the acute viral knockdown of STAT3. **C** STAT3 immunoreactivity in the DR was used to represent the efficiency of the viral knockdown strategy. STAT3 fluorescence signal intensity was quantified within the ROI (white rectangle) in the DR of AAV-Cre- vs. AAV-GFP-injected mice to (representative 4 × 5 stitched 60X images; scale bar: 100 µm). White dashed boxes mark the detailed area represented in (**D**), where single panel images (60X, scale bar: 50 µm) are shown. White arrows indicate examples of cells which are AAV-Cre or AAV-GFP transfected and have significant STAT3 immunoreactivity. These cells appear less numerous in the AAV-Cre group. **E** Quantification of overall STAT3 fluorescence intensity in the ROI confirmed Cre-mediated knockdown of STAT3 protein expression in the DR of the AAV-Cre group compared to AAV-GFP controls. **F** Transfection efficiency (DAPI + /GFP + coexpression vs. total DAPI + in ROI) and neurotropism (NeuN + /GFP + coexpression vs. total NeuN+ in ROI) were quantified in the DR of AAV-Cre injected mice. Behavioural evaluation of AAV-Cre mice did not reveal any differences in (**G**) locomotor activity in the open field or (**H**) sucrose preference in the SPT. **I** AAV-Cre mice showed significantly reduced latency to feed during the NSF while (**J**) their body mass, (**K**) the extent of weight loss due to food restriction, and (**l**) the amount of food consumed post-NSF was not impacted by DR STAT3 knockdown. **M** AAV-Cre mice displayed significantly reduced immobility in the FST when compared to the AAV-GFP group. **N** In contrast to AAV-GFP mice, AAV-Cre mice showed virtually no locomotor sensitisation to *d*-amphetamine. A.U.: arbitrary units; DR: dorsal raphe; FST: forced swim test; NSF: novelty-suppressed feeding; OFT: open field test; ROI: region of interest; SPT: sucrose preference test; STAT3: signal transducer and activator of transcription 3. All data are presented as mean ± SD. *p < 0.05.

In an attempt to disentangle possible developmental effects of STAT3 deletion from short-term or acute effects, we proceeded with a viral approach in order to knock down STAT3 in the DR of adult mice using AAV-GFP (control group) or AAV-Cre (STAT3 knockdown) in *Stat3*^fl/fl^ mice (Fig. 4A, B). STAT3 expression levels in the DR were quantified by comparing fluorescence intensities of total STAT3 immunoreactivity within the ROI (Fig. 4C), which confirmed STAT3 expression to be lower in the AAV-Cre group (Fig. 4D, E; *t*_8_ = 2.385; *p* = 0.044; *n* = 5/group). NeuN and DAPI immunoreactivity were employed to assess the average efficiency of viral transfection and neural tropism in the AAV-Cre group (Fig. 4F; *n* = 5–7/experiment). Behavioural experiments focused on paradigms in which genotype effects in the STAT3 KO experiments were observed. Similar to our observations in the STAT3 KO experiments, viral-mediated STAT3 knockdown did not lead to any alterations in baseline locomotor activity in the OFT (Fig. 4G; *t*_15_ = 1.164; *p* = 0.263; *n* = 8–9/group). Likewise, hedonic behaviour in the SPT was unchanged in AAV-Cre-injected mice compared to controls (Fig. 4H; *t*_15_ = 0.561; *p* = 0.583; *n* = 8–9/group). However, also mirroring STAT3 KO behaviour, AAV-Cre-injected mice exhibited shorter latencies to feed in the NSF (Fig. 4I; *t*_14_ = 2.767; *p* = 0.015; *n* = 8/group) than the AAV-GFP group. We controlled for basic differences in metabolism and appetite which may impact this behavioural trait by comparing body weight (Fig. 4J; *t*_14_ = 0.298; *p* = 0.770; *n* = 8/group), relative weight loss after food restriction (Fig. 4K; *t*_14_ = 1.163; *p* = 0.264; *n* = 8/group) and post-trial food consumption (Fig. 4L; *t*_9.168_ = 0.507; *p* = 0.624; *n* = 8/group), none of which were impacted by DR STAT3 knockdown. In the FST, the AAV-Cre group showed less immobility (Fig. 4M; *t*_15_ = 2.395; *p* = 0.030; *n* = 8–9/group), once again reminiscent of the results obtained in germline genetic STAT3 KO mice. Locomotor activity changes in response to amphetamine administration were significantly blunted in the AAV-Cre group when compared to AAV-GFP mice, phenocopying STAT3 KO behavioural performance (Fig. 4N; STAT3 knockdown – *F*_1,15_ = 7.412; *p* = 0.016; sensitisation – *F*_7,105_ = 27.180; *p* < 0.0001; STAT3 knockdown x sensitisation – *F*_7,105_ = 7.402; *p* < 0.0001; *n* = 8–9/group).

## Discussion

We here sought to systematically investigate the relevance of STAT3 in the midbrain serotonergic system for behavioural elements relevant to psychopathology. Integrating behavioural, electrophysiological and molecular units of analysis, we aimed to shed light on the functional consequences following the disruption of STAT3 signalling in the DR under consideration of neurodevelopmental aspects.

We identified serotonergic STAT3 as a modulator of DR neuronal excitability, determinant of behavioural reactivity and as a molecular switch for a transcriptional program determining intercellular communication with relevance to psychopathology.

In the first step, we focused on neuronal activity in serotonergic cells and revealed an impact of STAT3 deletion on the function of serotonergic cells reflected in increased 5-HT firing activity in KO DR neurons in vivo. This finding not only provided a first proof that the transcriptional program orchestrated by STAT3, respectively by the loss thereof, controls serotonergic cell activity but also indicated possible consequences at the behavioural level. Indeed, the “serotonergic vulnerability” theory postulates that the neurobiological correlate of increased susceptibility to environmental stressors could be a disrupted and therefore functionally dampened 5-HT system [61–63], as differences in 5-HT tone and 5-HT neuromodulatory control over other neurotransmitter systems are proposed to predominantly regulate how an individual reacts in adverse situations [64]. Our subsequent behavioural characterisation of STAT3 KO mice revealed a remarkably close confirmation of the expected phenotype. In the SPT, which is conducted in the home cage and does not involve additional adverse conditions, hedonic behaviour was unchanged in KO mice. On the contrary, in the NSF where the animal has to resolve an internal conflict between food drive and the stressful surroundings and in the FST where the inescapability of the situation drives active versus passive (immobility) behavioural choices [65], the phenotype of STAT3 deletion reflected a bias for reduced behavioural reactivity and increased proactive coping. Importantly, differences in behavioural response styles such as these are thought to represent main determinants of stress resilience, and by extension the propensity to develop psychiatric symptoms under adverse conditions [66, 67].

Our data are also well in agreement with existing literature. It has been reported that systemic blockade of STAT3 by the small molecule stattic reduced immobility in the FST [5] and that genetic STAT3 inactivation in microglia led to a reduction in immobility in the tail suspension test and FST, but neither impacted performance in the SPT at baseline nor in response to restraint stress [28], suggesting that STAT3 deletion induces a stress-resilient phenotype. Moreover, microglial deletion of STAT3 was additionally associated with increased electrophysiological activity in slices from medial prefrontal cortex of these mice [28]. This is reminiscent of our finding of increased in vivo spontaneous firing in the DR of serotonergic STAT3 KO and supports a role for STAT3-regulated transcriptional networks in neurotransmission. Further support for the involvement of STAT3 in psychopathology is derived from studies implicating it in the action of multiple pharmacological and non-pharmacological psychiatric treatment approaches in experimental model systems [27, 29, 31, 32, 68–73]. Importantly, several important STAT3 upstream regulators may converge on activation of STAT3, including among others cytokine receptors, receptor tyrosine kinases and non-receptor tyrosine kinases as well as G-Protein coupled receptors and small regulatory RNAs (reviewed in [24]), all of which are critically involved in neural function underlying “normal” behavioural processes and their deviation in psychopathology (reviewed in [74]).

The STAT3 KO line used in the present study constitutes a functional STAT3 knock-out with a Cre-dependent deletion of exons 18, 19 and 20 harbouring the coding region for the SH2 domain, essential for STAT3 function [38]. The immunohistochemical characterization of the STAT3 KO line illustrated the pattern of STAT3 deletion, albeit the complete absence of STAT3 cannot be unequivocally demonstrated in every serotonergic cell. This aspect may be a consequence of the efficacy of the specific genetic strategy in our hands and should be taken into consideration for the further evaluation of the results.

qRT-PCR analysis confirmed the significant decrease of this specific region of the STAT3 transcript in DR tissue of STAT3 KO versus control animals. Since bulk tissue of the DR and not serotonergic cells only was used as input for the qRT-PCR, the observed decrease is only modest, but highly significant.

The transcriptional patterns revealed by RNA-Seq in STAT3 KO DR tissue are not only surprisingly rich in processes and pathways relevant to psychopathology, at the single gene and the gene set level, but also corroborate the relevance of STAT3 for intercellular communication, with a striking 13 out of the 16 significantly enriched categories and pathways across both analyses related to neurotransmission-relevant processes. These include genes pertinent to ion transport, the synaptic vesicle cycle, calcium signalling, and neurotransmitter transport. The significant increase in spontaneous firing of DR 5-HT neurons in STAT3 KO also ties in perfectly with the reported transcriptional changes in pathways mediating synaptic transmission and neuronal excitability.

Guided by the RNA-Seq analysis, we next extended the behavioural examination to paradigms pertinent to amphetamine addiction, which are therefore also relevant to behavioural constructs of the positive valence system. The blunted sensitisation response of STAT3 KO mice is notable considering the previously discussed evidence for reduced stress sensitivity, as the interrelationship between stress and psychostimulant action is solidly documented and cross-sensitisation between repeated exposure to amphetamine and stress is well-described [75–79]. Notably, the observation that serotonergic STAT3 did not affect the rewarding properties of amphetamine as evaluated in the CPP test is also strengthened by the unaltered hedonic responses in the SPT displayed by these mice.

An important aspect for the interrogation of the involvement of STAT3 in psychopathology is the potential relationship of the behavioural phenotype of STAT3 KO mice to neurodevelopmental processes. As the acute viral-mediated, location-specific knockdown of STAT3, which indicated that short-term depletion of STAT3 in the DR had comparable effects to the constitutive knockout of STAT3 in 5-HT neurons, we concluded that developmental mechanisms are likely not involved.

Taken together, by integrating behavioural with cellular and molecular units of analysis we are in a position to propose a role for STAT3 in the DR in the display of behavioural elements relevant to psychopathology. Collectively, the herein presented data allow for the postulation of a tentative model in which serotonergic STAT3 acts as a molecular gate to tune behavioural reactivity. According to this model, the transcriptional program controlled by STAT3 in the DR constitutes a mechanism regulating sensitivity and behavioural response to aversive conditions, possibly via modulation of central serotonergic function. The diversity of stimuli capable of inducing STAT3 activity further highlights the context-dependent pertinence of our findings, which collectively postulate a mechanistic involvement of serotonergic STAT3 in the regulation of behavioural reactivity, with relevance for psychopathology across boundaries of distinct disease entities.

## Supplementary information

Suppl Materials and Methods

Suppl Fig 1

Suppl Table 1

Suppl Table 2

Suppl Table 3A

Suppl Table 3B

Suppl Table 3C

Suppl Table 3C
