# Supplementary material for: STAT3 in the dorsal raphe gates behavioural reactivity and regulates gene networks associated with psychopathology
Source: Mol Psychiatry. 2020 Oct 12;26(7):2886–99. doi: 10.1038/s41380-020-00904-2 (PMC8505245; doi:10.1038/s41380-020-00904-2)
Supplement: Supplementary file 1 — Suppl Materials and Methods [file 41380_2020_904_MOESM1_ESM.docx]

Reisinger SN *et al.* STAT3 in the dorsal raphe gates behavioural reactivity and regulates gene networks associated with neuropsychiatric disorders

**SUPPLEMENTARY MATERIALS AND METHODS**

1. **Animals**

Conditional STAT3 knockout mice *Sert*^Cre/+^; *Stat3*^fl/fl^ (“KO” hereafter) and littermate *Sert*^Cre/+^; *Stat3*^fl/+^ (“control” hereafter) were bred on a C57 x 129 hybrid background by crossing *Sert*^+/+^; *Stat3*^fl/fl^ females with *Sert*^Cre/+^; *Stat3*^fl/+^ males. Founder pairs were purchased from commercial providers (B6.129S1-Stat3<tm1Xyfu>/J; Charles River, Sulzfeld, GER; B6.129(Cg)-Slc6a4<tm1(cre)Xz>/Cnrm; European Mouse Mutant Archive, Monterotondo Scalo, Rome, ITA).
Littermate Sert^+/+^; *Stat3*^fl/fl^ animals of the same genetic line were employed for viral knockdown experiments. All mice were kept in a temperature-controlled colony room (22 ± 1°C) and provided with food and water *ad libitum*. Animals were maintained on a 12/12 hour light/dark cycle (with lights on at 7:00 a.m., 20 lx inside the cages). Male and female littermate mice were used for all experiments and were 8-16 weeks old at onset of experiments.
All *in vivo* experiments were performed during the light phase of the cycle and mice were single-housed for one week prior to the onset of testing. The experimenter was blind to the genotypes of mice during all behaviour testing and electrophysiological recordings until data analyses.

All animal experiments were conducted strictly adhering to the European Communities Council Directive of September 22, 2010 (2010/63/EU) and only after approval by the national authorities (Austrian Federal Ministry of Education, Science and Research, no. BMBWF-66.009/0175-V/3b/2019). All experimental protocols have been designed in consideration of and in accordance with the “3R- principles” (<https://www.nc3rs.org.uk/the-3rs>).

1. ***In vivo* electrophysiology of DR 5-HT neurons**

Mice were deeply anaesthetised using ketamine/xylazine (Ketanest, 100 mg/kg, Pfizer, Vienna, AT; Rompun, 20 mg/kg, Bayer, Vienna, AT; i.p., both 10 ml/ kg) and head-fixed in a stereotactic frame for rodents (Kopf instruments, Tujunga, CA, US). *In vivo* recordings of DR 5-HT neurons were essentially conducted as previously reported [1] . Briefly, borosilicate glass pipettes (resistance: 5–7 MΩ) were obtained using a horizontal micropipette puller (Sutter Instrument, Novato, CA, US) and filled with artificial cerebrospinal fluid (aCSF: 125 mM NaCl, 2.5 mM KCl, 1.25 mM NaH_2_PO_4_, 25 mM *d*-(+)glucose, 25 mM NaHCO_3_, 1 mM MgCl_2_, 2 mM CaCl_2_; pH=7.4).
The DR was targeted at Bregma -4.5 mm (rostrocaudal axis) [2], however this coordinate was adjusted for each mouse using the conversion factor *f*, which takes into account differences in skull size and by extension brain size, and which is simply the ratio [3] between measured and published Bregma to Lambda (B-λ) distances: $\text{ }\text{f }\text{= }\frac{\text{ }B-\lambda(measured)}{B-\lambda(in Atlas)}$.
Following the calculation of *f*-adjusted coordinates, a hole was drilled into the skull at the correct rostrocaudal location. The electrode was lowered into the brain in steps of 0.5 mm (dorsoventral axis) until the recognition of characteristic serotonergic firing properties (slow frequency: ~0.8-2.5 Hz; long duration spikes: ~0.8-1.2 ms) [4–6]. Upon identification of serotonergic action potentials, spontaneous activity was recorded for at least 4 minutes.

An Axoclamp-2B amplifier (Axon Instruments, Union City, CA, US) in bridge mode was used to obtain extracellular recordings, and analogue-to-digital data conversion was done using the Digidata-1440 interface (Axon Instruments). Data processing and analysis were performed using the software package pClamp-10 and Clampfit, respectively (both Axon Instruments).

1. **Behaviour**

Sample sizes were first determined according to our own and others’ published results of comparable behavioural studies [1, 7, 8]. Since the analysis of the STAT3 KO behaviours revealed no *genotype × sex* interactions, males and females were collapsed into a single group of each genotype.

Prior to the start of behavioural testing, mice were habituated to the experimenter by handling for one minute on two non-consecutive days. On testing days all mice were transferred to the experimental room at least 45 minutes prior to the start of behavioural analyses.

*Sucrose preference test*

For the sucrose preference test (SPT) [1], mice were habituated to the sucrose solution (2% in tap water, Sigma-Aldrich, Vienna, AT) over a training period of 48 hours starting three days before the test day. After a switch back to normal tap water for 6 hours, mice were then food- and water-restricted for 18 hours prior to the test. During the 3-hour test period mice were given sucrose solution and water in identical bottles in a forced choice paradigm and liquid consumption from each bottle was determined. Bottle sides were alternated to avoid side bias. The relative sucrose preference (% of total liquid consumption) for each animal was calculated and used as indicator of hedonic behaviour [9].

*Novelty-suppressed feeding test*

Depression-related anxiety was examined in the NSF [1], in which mice (food-restricted for 24 hours prior to testing) were placed in the corner of brightly-lit arena filled with corn-cob bedding material (ca. 30 × 50 cm^2^; illuminance in centre: 800 lx), at the centre of which a single pellet of food was placed. The time from trial start until the mouse took the first bite of the pellet (latency to feed, maximum time allowed: 15 minutes) was recorded and used as relevant parameter of hyponeophagia [10]. To control for potential metabolic biases, body weight prior to the start of testing (g), body mass loss during food restriction (%) and home cage food consumption (during 5 minutes immediately after testing) of a single pre-weighed pellet were additionally analysed.

*Forced swim test*

The forced swim test (FST) was carried out using automated movement tracking software (VideoTrack v3, Viewpoint, Champagne au Mont d’Or, FR) [1]. Mice were placed into a beaker filled with tap water (23 ± 1 ˚C) for 6 minutes and recorded, with average immobility (%, defined as the absence of movement bar those necessary for floating) during the final 4 minutes evaluated and used as indicator of coping style when faced with inescapable stressors [11].

*Open field test*

The open field test (OFT) was performed to measure spontaneous locomotor activity (total distance travelled, m) upon placement into a novel environment [1]. Arenas (27.3 x 27.3 cm^2^; illuminance 300 lx) fitted with laser beams for automatic tracking of the animals’ movements were used in combination with the accompanying analysis software (Activity Monitor v5, MedAssociates, Fairfax, Vermont, US). Trials lasted 5 minutes and mice were placed into the lower left corner of the arena, initiating an automatic start.

*Light-dark box test*

Anxiety-like behaviour was evaluated using the light-dark box test (LDB) in which an opaque Plexiglas insert was placed into one side of the open field apparatus described above, with a small entrance facing the middle of the arena (illuminance of dark compartment < 5 lx; light compartment 300 lx) [1]. Mice were placed inside the dark boxes to start the 10-minute trial. Automated software-based analysis software (MedAssociates) provided the time spent in the aversive light zone (%) as indicator of anxiety-like behaviour [12].

*Elevated plus maze*

Anxiety-like behaviour in the elevated plus maze (EPM) was determined by placing the mice in the centre of a plus-shaped maze with two opposing closed arms (illuminance 10 lx, walls ~ 20 cm) and two opposing open arms, with the entire maze being elevated about 50 cm off the ground. Mice were automatically tracked using a camera and the software Videotrack (Viewpoint) during a 5-minute trial. Time spent in the aversive open arms (%) was calculated by the software and was used to determine levels of anxiety-like behaviour [13].

*Rotarod*

Motor coordination was tested using an automatic Rotarod (RR) system for rodents (MedAssociates) consisting of a rotating drum (increasing in speed from 4 to 40 RPM over 6 minutes) with separator walls and fitted with laser beam detectors [1]. Latency to fall was automatically recorded. Mice underwent 3 trials, and the average latency to fall off the Rotarod (s) was calculated.

*Amphetamine sensitisation*

Locomotor sensitisation (as % change in distance travelled from day 1/baseline) to *d-*amphetamine after repeated daily injection was evaluated in the open field (apparatus as above) for 30 minutes after i.p. injection of *d-*amphetamine (2 mg/kg free base in NaCl 0.9 %, 10 ml/kg volume) (protocol adapted from [14]). One day prior to the start of experiments (day 0), mice were injected with NaCl (0.9 %, 10 ml/kg) in the animal housing facility to habituate them to the injection procedure. The next day (day 1), they were subjected to the open field for the first time after saline injection for the determination of baseline locomotor activity. On all following days (days 2-7, day 15), mice were injected with *d-*amphetamine immediately prior to being placed in the open field. As outlined above, mice were tracked using an automated system and accompanying software (MedAssociates).

*Conditioned place preference*

CPP was evaluated using a biased experimental paradigm [15]. On the first day (day 1) mice were allowed to roam freely in a two-compartment box (floor compartment A: grid; compartment B: bars) fitted into the automated open field system described above. Side preference (%) on day 1 was calculated for each mouse for the determination of drug-compartment pairings in subsequent training sessions, during which compartments were separated by a sliding door and mice were thus restricted to one compartment. On days 2, 4 and 6 mice were injected with *d*-amphetamine (5 mg/kg free base, in NaCl 0.9 %, 10 ml/kg volume i.p.) and placed into the less-preferred compartment for 30 min, while on days 3, 5 and 7 they were placed into the preferred compartment after injection with saline solution (0.9 %, 10 ml/kg volume i.p.). On day 8, the compartment-separating door was removed again and mice were tracked while they were able to freely choose between compartments. Side preference (%) was calculated and compared between groups to evaluate differences in associative learning.

1. **Drugs**

*D*-amphetamine sulphate (GlaxoSmithKline, London, GBR) was dissolved in physiological saline (0.9%) for administration during amphetamine sensitisation (dosage 2 mg/ kg) and CPP (dosage 5 mg/ kg; injection volume 10 ml/ kg) experiments. To obtain the indicated free-base concentrations, a conversion factor of 1.36 was applied to calculate the correct amount of *d*-amphetamine sulphate to be used.

1. **Immunohistology**

Mice were anaesthetised (ketamine: Ketanest, 100 mg/kg, Pfizer; xylazine: Rompun, 40 mg/kg, Bayer; i.p., both 10 ml/ kg), then transcardially perfused with physiological saline (0.9% NaCl) followed by 4% paraformaldehyde in PBS before extracting the brain and fixing it for 24 hours in 4% paraformaldehyde at 4 °C. After a further 48 hours in 30% sucrose at 4 °C, brains were embedded in O.C.T. (Sakura Finetek), frozen and stored at -80 °C before cutting and collecting 30 µm sections of the target area, the dorsal raphe nuclei (DR) (coordinates approximately -4.0 mm to -5.2 mm [2]) using a Leica CS1950 cryotome (Leica Microsystems). Free-floating sections were stored in cryoprotectant solution (30 % glycerol, 30% ethylene glycol, 40% PBS 1X, Sigma-Aldrich) at -20 °C prior to the immunostaining procedure. For 5-HT/STAT3 only, brain sections were subjected to antigen retrieval procedure, involving the incubation of free-floating sections in 10 mM sodium citrate buffer (pH= 8.5) heated to 80 °C for 30 minutes, then for a further 30 minutes while cooling to room temperature. Standard immunofluorescence protocols were then applied for double 5-HT/STAT3, Iba1/STAT3, GFAP/STAT3, STAT3 and NeuN stainings. Briefly, sections were washed in Tris buffered saline with Triton-X 0.03% (TBST) and blocked for 30 min using 5 % donkey serum (5-HT/STAT3), 1% bovine serum albumin (Iba1/STAT3, GFAP/STAT3, STAT3) or 3% goat serum (NeuN) in TBST. Sections were incubated with the primary antibodies in TBST (goat anti-5-HT, ab66047, Abcam, 1:200; combined with rabbit anti-STAT3, sc-7179, Santa Cruz Biotechnology, 1:100; *or* rabbit anti-Iba1, 019-19741, Fujifilm Wako Pure Chemical Corporation, 1:1000; combined with mouse anti-STAT3, ab119352, Abcam, 1:100; *or* rabbit anti-GFAP, G4546, Sigma-Aldrich, 1:500; combined with mouse anti-STAT3, ab119352, Abcam, 1:100, *or* mouse anti-STAT3, ab119352, Abcam, 1:100; *or* mouse anti-NeuN, MAB377, Chemicon/ Sigma-Aldrich, 1:500) for 72 hours at 4° C on a shaker. After three washes, secondary antibody incubation (for 5-HT/STAT3: donkey anti-goat, Alexa 488, A11055, Invitrogen, 1:200, combined with donkey anti-rabbit, Alexa 594, A21207, Invitrogen, 1:500, 1.5 hours; for Iba1/STAT3: donkey anti-rabbit, Alexa 488, A11034, Invitrogen, 1:200, combined with goat anti-mouse, CF 594, SAB4600402, Merck, 1:500, 1.5 hours; for GFAP/STAT3: donkey anti-rabbit, Alexa 488, A11034, Invitrogen, 1:200, combined with goat anti-mouse, CF 594, SAB4600402, Merck, 1:500, 1.5 hours; for NeuN: goat anti-mouse, CF 594, SAB4600402, Merck, 1:1000, 1 hour) was performed at room temperature on a shaker. Following three further washes, sections were mounted onto glass slides using fluorescence mounting medium (Dako Denmark A/S, Glostrup, DNK) and images were acquired using a Nikon A1 confocal microscope with 20X, 60X or 100X objectives and the software NIS-Elements AR (version 5.02.01, Nikon Instruments Inc., Tokyo, JP).

1. **Quantitative analysis of immunohistology**

STAT3 fluorescence intensity was quantified using the software ImageJ [16] according to a published procedure [17]. Briefly, for 5-HT/STAT3, large images consisting of 4 by 5 60X panels for each channel (5-HT: green; STAT3: red) were obtained, centred on the DR area *(see main article text for details)*. Since microglia and astrocytes are far smaller than serotonergic neurons, less abundant and more dispersed in the DR, 100X multichannel images were acquired for Iba1/STAT3 and GFAP/STAT3 (Iba1 or GFAP: green; STAT3: red). For quantification of STAT3 immunoreactivity in serotonergic neurons (5-HT/STAT3), microglia (Iba1/STAT3) and astrocytes (GFAP/STAT3), areas of the images where a signal was detected in the green-channel image (i.e. 5-HT-, Iba1- or GFAP-positive cells) were selected and the green-channel images were used to create individual selection masks (“ROIs) applied to the STAT3 channel to restrict the analysis to relevant cells. STAT3 signal intensity was measured in red-channel 8-bit images within the pre-defined ROI, and intensity values normalised to image size were used for statistical analysis.

In the case of STAT3 quantification and quantification of viral transfection efficiency for the viral knockdown experiments, the ROI was defined as a rectangle (approx. 700 × 800 µm^2^, directly ventral to cerebral aqueduct at midline) corresponding to the DR. Overall STAT3 fluorescence signal intensity in this ROI was measured using ImageJ and compared between groups to confirm the knockdown of STAT3 protein expression in the DR area of AAV-Cre-injected animals.

For analysis of transfection efficiency, sections from the DR of the AAV-Cre-injected cohort were stained for NeuN and DAPI. Within the rectangular ROI, defined as described above, GFP-positive cells (i.e. virally transfected cells) were counted and related to the total number of DAPI-positive cells (transfection efficiency, %) or to the number of NeuN-positive cells (neurotropism, %) observed.
File conversion to .tif was performed with the program ImageJ [16] and the open-source software QuPath was employed for cell counting analyses [18].

1. **Brain extraction**

For RNA and protein sample collection, mice were killed by cervical dislocation, whole brains were extracted and rapidly embedded in O.C.T. medium (Sakura Finetek, Staufen im Breisgau, DE). These were rapidly placed onto a rack in a N2-filled box, well clear of the liquid portion, to flash-freeze the brains and then stored at -80 ˚C until further processing. The dorsal raphe nucleus (DR) area was collected from four 300 µm-thick coronal sections (Bregma -4.0 mm to Bregma -5.2 mm [2]) obtained using a Leica cryotome (Leica CS1950, Leica Microsystems, Wetzlar, DE) set to -8 °C. A microdissection knife was used to dissect a square area (1.5 x 1.5 mm^2^) directly below the aqueduct in each section, containing the DR including lateral wings and ventromedial portion [2]. The tissue was stored in 400 µl RNALater (ThermoFisherScientific, Vienna, AT) at -20 ˚C until further processing.

1. **RNA extraction**

RNA extraction was performed using miRNEasy Mini Kit (Qiagen, Venlo, NL) by following the manufacturer’s protocol (“Quick start guide”). Briefly, tissue was removed from RNAlater and placed into 700 µl Qiazol (Qiagen) and mechanically disrupted until the homogenate was clear. This mix was incubated at room temperature (RT) for 5 min before addition of chloroform, with a further incubation period of 3 min. After centrifugation (15 min, 12000 G, 4 °C), the upper phase was transferred and RNA was precipitated with 1.5 × the sample volume in ethanol (96 %). Provided columns and collection tubes were used to retain the precipitate prior to washing the filter with the provided wash buffers (RWT once, RPE twice). At each of these steps, the flowthrough after a quick centrifugation step was discarded. Finally, RNA was eluted from the column filter using 40 µl of RNAse-free water.

1. **RNA-Seq and bioinformatic analysis**

RNA samples used for RNA-Seq were checked for quality using a Bioanalyzer (Agilent Technologies, Santa Clara, CA, US) and showed RNA Integrity Numbers (RIN) above 7.0. Library preparation and sequencing was performed by the Core facility Genomics, Medical University of Vienna, as previously described elsewhere [19]. Briefly, we prepared sequencing libraries using the NEBNext® Ultra™ II RNA Library Prep Kit (New England Biolabs, Ipswich, MA, US) by following the manufacturer’s protocol, and sequencing was carried out on the Illumina NExtSeq500 platform (Illumina, San Diego, CA, US) in 75 bp single-read mode.
Data was initially analysed on the Illumina Basespace platform (Illumina). Briefly, RNA-Seq data were mapped to the Mus musculus/mm10 assembly of
the murine genome using STAR Aligner [20].
Differential gene expression was analysed using DESeq2 [21]. Raw *p* values were adjusted to obtain *q* values, corrected for multiple comparisons by
applying the Benjamini-Hochberg method and a false discovery rate of 5%.
Raw and processed data are available online at the Gene Expression Omnibus (GEO) data repository of the National Center for Biotechnology Information (NCBI) under the accession number GSE146101.

Differentially expressed genes (DEG) were subjected to *post hoc* Venn (Venny 2.1.0 [22]) and gene list enrichment analysis with the online tool Enrichr [23, 24], focussing on gene ontologies (GO) relating to biological processes [25, 26] and KEGG pathways (Kyoto Encyclopedia of Genes and Genomes [27]). The top 5 significant gene sets or KEGG pathways (ranked by adjusted *p* value according to Enrichr) are reported. For Venn analysis of the DEG set, a list of confirmed STAT3 targets was obtained from ChIP-Atlas, a database of ChIP-Seq data [28], while lists of genes and variants associated with MDD, SCZ and BP were obtained from the online database DisGeNet [29].

1. **Quantitative Real Time-PCR**

The procedure was carried out as previously described [1]. For cDNA synthesis using the DyNamo cDNA Synthesis Kit (ThermoFisherScientific), 450 ng of DR RNA were used per sample and the manufacturer’s protocol was followed exactly. Controls lacking RNA template input or reverse transcriptase were included to check for contamination of samples and reagents.
qRT-PCR was carried out using a Bio-Rad CFX Connect PCR cycler (Bio-Rad, Hercules, CA, US) and Go-Taq qPCR Master Mix for dye-based qPCR (Promega, Madison, WI, US). STAT3 and β-actin primers were obtained from Invitrogen (Vienna, AT) and diluted to a working concentration of 20 µM. Sequences are listed in Supplementary Table 2. All reactions were performed in duplicates, and semi-quantitative analysis of STAT3 expression vs. β-actin expression was carried out using the ΔΔC(t) method [30].

1. **Viral STAT3 knockdown STAT3**

A timeline of experimental procedures is provided in Figure 4B.

Male and female STAT3^fl/fl^ mice were deeply anaesthetised with isoflurane (4-4.5% for induction; 1.7-2.5% for maintenance; Forane, Baxter, Deerfield, IL, US) and head-fixed in a stereotactic frame (Kopf Instruments). A microsyringe (Hamilton, Reno, NV, US) controlled by a microinjection pump (Longer Precision Pump Co., Hebei, CHN) was used to infuse 0.5 µl of AAV-Cre (pAAV.CMV.HI.eGFP-Cre.WPRE.SV40, Addgene viral prep #105545-AAV5) or AAV-GFP (pAAV.CMV.PI.EGFP.WPRE.bGH; Addgene viral prep #105530-AAV5; produced by James M. Wilson; titer: 10^13^  genome copies/ ml) over 8 minutes at each of the following coordinates relative to Bregma (adjusted for each mouse using a conversion factor *f* as outlined above [3]): (a) x= 0.0, y= -4.5 , z= -2.9; (b) x= 0.0, y= -4.5, z= -2.7. These coordinates were chosen to target the centre of the dorsal raphe nucleus (DR). Two injection sites were targeted due to the high white matter density of the DR which is reported to hamper the spread of viral particles [31]. The AAV5 serotype was chosen due to its ability to transfect neurons stably [32]. A pause of 2 minutes before and 5 minutes after each syringe discharge was observed to allow for brain tissue to settle, and to avoid spreading the virus upon syringe retraction. After suturing, mice received post-operative analgesia (tramadol, dosage 60 mg/ kg, volume 10 ml/ kg, i.p.; Tramal, Grünenthal, Aachen, DE; carprofen in tap water, dosage 7.5 mg/ ml, p.o. for 3 days, Rimadyl, Zoetis, Parsippany-Troy Hills, NJ, US) and were allowed to recover for two weeks before the start of the first experimental procedures (SPT training).

Behavioural tests were carried out as detailed above, and in the following order: SPT, NSF, OFT, FST, amphetamine sensitisation. Upon termination of behavioural experiments, mice were transcardially perfused with 4% paraformaldehyde as detailed above for immunofluorescence procedures, and 30 µm DR sections were collected, mounted using fluorescence mounting medium (Dako Denmark A/S, Glostrup, DK) and examined for the presence and overall spread of GFP+ cells in the DR to determine viral gene expression in each animal. Following this procedure, we excluded 3-4 out of 12 mice per group in which the injection site or the viral spread was deemed unsuitable.

1. **Statistical analysis and graphical representations**

GraphPad Prism 7.0 (GraphPad Software Inc., San Diego, CA, US) was used for all statistical analyses and preparation of graphs. Two-tailed, two-sample Student’s *t* test was applied wherever two groups were being compared, as data were mostly normally distributed. Where groups did not show equal variances (as calculated by *F*-test), a Welch correction was applied. In the case of a repeated measures design, such as for amphetamine sensitisation analysis, repeated-measure ANOVA was applied. All data is shown as mean ± standard deviation (SD), and individual values are represented graphically.
Statistical outliers falling outside the interval mean ± 2 SD were pre-excluded from analyses to avoid skewing the data, as this interval represents >95% of values around the mean of a normally distributed dataset.

Python was used to prepare the volcano plot (Figure 3A) and heatmap of viral transduction in AAV-Cre animals (Figure 4A).

**SUPPLEMENTARY FIGURE LEGEND**

**Supplementary Figure 1.**

Microglial and astrocytic STAT3 expression in the DR area is unaltered in STAT3 KO animals. (A, B) Examination of STAT3 immunoreactivity within Iba1/ GFAP-positive cells (representative images: 100X, scale bar: 20 µm; ROI: white outlines used as masks in STAT3 channel) and (C, D) accompanying quantification of STAT3 fluorescence intensity revealed no differences in STAT3 expression between STAT3 KO and control mice in the DR. A.U.: arbitrary units; DR: dorsal raphe; GFAP: glial fibrillary acidic protein; Iba1: ionized calcium-binding adapter molecule 1; KO: knockout; STAT3: signal transducer and activator of transcription 3. All data are presented as mean ± SD.

**REFERENCES**

1. Reisinger SN, Kong E, Molz B, Humberg T, Sideromenos S, Cicvaric A, et al. Flotillin-1 interacts with the serotonin transporter and modulates chronic corticosterone response. Genes Brain Behav. 2018:e12482.

2. Paxinos G. Paxinos and Franklin’s the mouse brain in stereotaxic coordinates /. 4th ed. Amsterdam : Boston :; 2013.

3. Athos J, Storm DR. High precision stereotaxic surgery in mice. Curr Protoc Neurosci. 2001;Appendix 4:Appendix-4A.

4. Gobbi G, Murphy DL, Lesch K, Blier P. Modifications of the serotonergic system in mice lacking serotonin transporters: an in vivo electrophysiological study. J Pharmacol Exp Ther. 2001;296:987–995.

5. Lira A, Zhou M, Castanon N, Ansorge MS, Gordon JA, Francis JH, et al. Altered depression-related behaviors and functional changes in the dorsal raphe nucleus of serotonin transporter-deficient mice. Biol Psychiatry. 2003;54:960–971.

6. Allers KA, Sharp T. Neurochemical and anatomical identification of fast- and slow-firing neurones in the rat dorsal raphe nucleus using juxtacellular labelling methods in vivo. Neuroscience. 2003;122:193–204.

7. Reisinger SN, Wanek T, Langer O, Pollak DD. PET imaging of the mouse brain reveals a dynamic regulation of SERT density in a chronic stress model. Transl Psychiatry. 2019;9:1–11.

8. Mosienko V, Bert B, Beis D, Matthes S, Fink H, Bader M, et al. Exaggerated aggression and decreased anxiety in mice deficient in brain serotonin. Transl Psychiatry. 2012;2:e122.

9. Der-Avakian A, Markou A. The neurobiology of anhedonia and other reward-related deficits. Trends in Neurosciences. 2012;35:68–77.

10. Samuels BA, Hen R. Novelty-Suppressed Feeding in the Mouse. In: Gould TD, editor. Mood and Anxiety Related Phenotypes in Mice: Characterization Using Behavioral Tests, Volume II, Totowa, NJ: Humana Press; 2011. p. 107–121.

11. Molendijk ML, de Kloet ER. Coping with the forced swim stressor: Current state-of-the-art. Behavioural Brain Research. 2019;364:1–10.

12. Bourin M, Hascoët M. The mouse light/dark box test. Eur J Pharmacol. 2003;463:55–65.

13. Walf AA, Frye CA. The use of the elevated plus maze as an assay of anxiety-related behavior in rodents. Nat Protocols. 2007;2:322–328.

14. Steinkellner T, Montgomery TR, Hofmaier T, Kudlacek O, Yang J-W, Rickhag M, et al. Amphetamine action at the cocaine- and antidepressant-sensitive serotonin transporter is modulated by αCaMKII. J Neurosci. 2015;35:8258–8271.

15. Prus AJ, James JR, Rosecrans JA. Conditioned Place Preference. In: Buccafusco JJ, editor. Methods of Behavior Analysis in Neuroscience, . 2nd ed.Boca Raton (FL): CRC Press/Taylor & Francis; 2009.

16. Schneider CA, Rasband WS, Eliceiri KW. NIH Image to ImageJ: 25 years of image analysis. Nat Methods. 2012;9:671–675.

17. Bankhead P. Analyzing fluorescence microscopy images with ImageJ · GitBook (Legacy). GitBook. https://legacy.gitbook.com/book/petebankhead/imagej-intro/details. Accessed 22 February 2020.

18. Bankhead P, Loughrey MB, Fernández JA, Dombrowski Y, McArt DG, Dunne PD, et al. QuPath: Open source software for digital pathology image analysis. Sci Rep. 2017;7:1–7.

19. Reisinger SN, Bilban M, Stojanovic T, Derdak S, Yang J, Cicvaric A, et al. Lmo3 deficiency in the mouse is associated with alterations in mood-related behaviors and a depression-biased amygdala transcriptome. Psychoneuroendocrinology. 2020;111:104480.

20. Dobin A, Davis CA, Schlesinger F, Drenkow J, Zaleski C, Jha S, et al. STAR: ultrafast universal RNA-seq aligner. Bioinformatics. 2013;29:15–21.

21. Love MI, Huber W, Anders S. Moderated estimation of fold change and dispersion for RNA-seq data with DESeq2. Genome Biology. 2014;15:550.

22. VENNY. OmicX. https://omictools.com/venny-tool. Accessed 10 November 2019.

23. Chen EY, Tan CM, Kou Y, Duan Q, Wang Z, Meirelles GV, et al. Enrichr: interactive and collaborative HTML5 gene list enrichment analysis tool. BMC Bioinformatics. 2013;14:128.

24. Kuleshov MV, Jones MR, Rouillard AD, Fernandez NF, Duan Q, Wang Z, et al. Enrichr: a comprehensive gene set enrichment analysis web server 2016 update. Nucleic Acids Res. 2016;44:W90–W97.

25. Ashburner M, Ball CA, Blake JA, Botstein D, Butler H, Cherry JM, et al. Gene ontology: tool for the unification of biology. The Gene Ontology Consortium. Nat Genet. 2000;25:25–29.

26. The Gene Ontology Consortium. The Gene Ontology Resource: 20 years and still GOing strong. Nucleic Acids Res. 2019;47:D330–D338.

27. Kanehisa M, Goto S. KEGG: kyoto encyclopedia of genes and genomes. Nucleic Acids Res. 2000;28:27–30.

28. Oki S, Ohta T, Shioi G, Hatanaka H, Ogasawara O, Okuda Y, et al. ChIP-Atlas: a data-mining suite powered by full integration of public ChIP-seq data. EMBO Reports. 2018;19:e46255.

29. Piñero J, Ramírez-Anguita JM, Saüch-Pitarch J, Ronzano F, Centeno E, Sanz F, et al. The DisGeNET knowledge platform for disease genomics: 2019 update. Nucleic Acids Res. https://doi.org/10.1093/nar/gkz1021.

30. Schmittgen TD, Livak KJ. Analyzing real-time PCR data by the comparative C T method. Nature Protocols. 2008;3:1101–1108.

31. Correia P, Matias S, Mainen Z. Stereotaxic Adeno-associated Virus Injection and Cannula Implantation in the Dorsal Raphe Nucleus of Mice. BIO-PROTOCOL. 2017;7.

32. Davidson BL, Stein CS, Heth JA, Martins I, Kotin RM, Derksen TA, et al. Recombinant adeno-associated virus type 2, 4, and 5 vectors: Transduction of variant cell types and regions in the mammalian central nervous system. Proc Natl Acad Sci U S A. 2000;97:3428–3432.
