# Supplementary figures and images for: STAT3 in the dorsal raphe gates behavioural reactivity and regulates gene networks associated with psychopathology
Source: Mol Psychiatry. 2020 Oct 12;26(7):2886–99. doi: 10.1038/s41380-020-00904-2 (PMC8505245; doi:10.1038/s41380-020-00904-2)

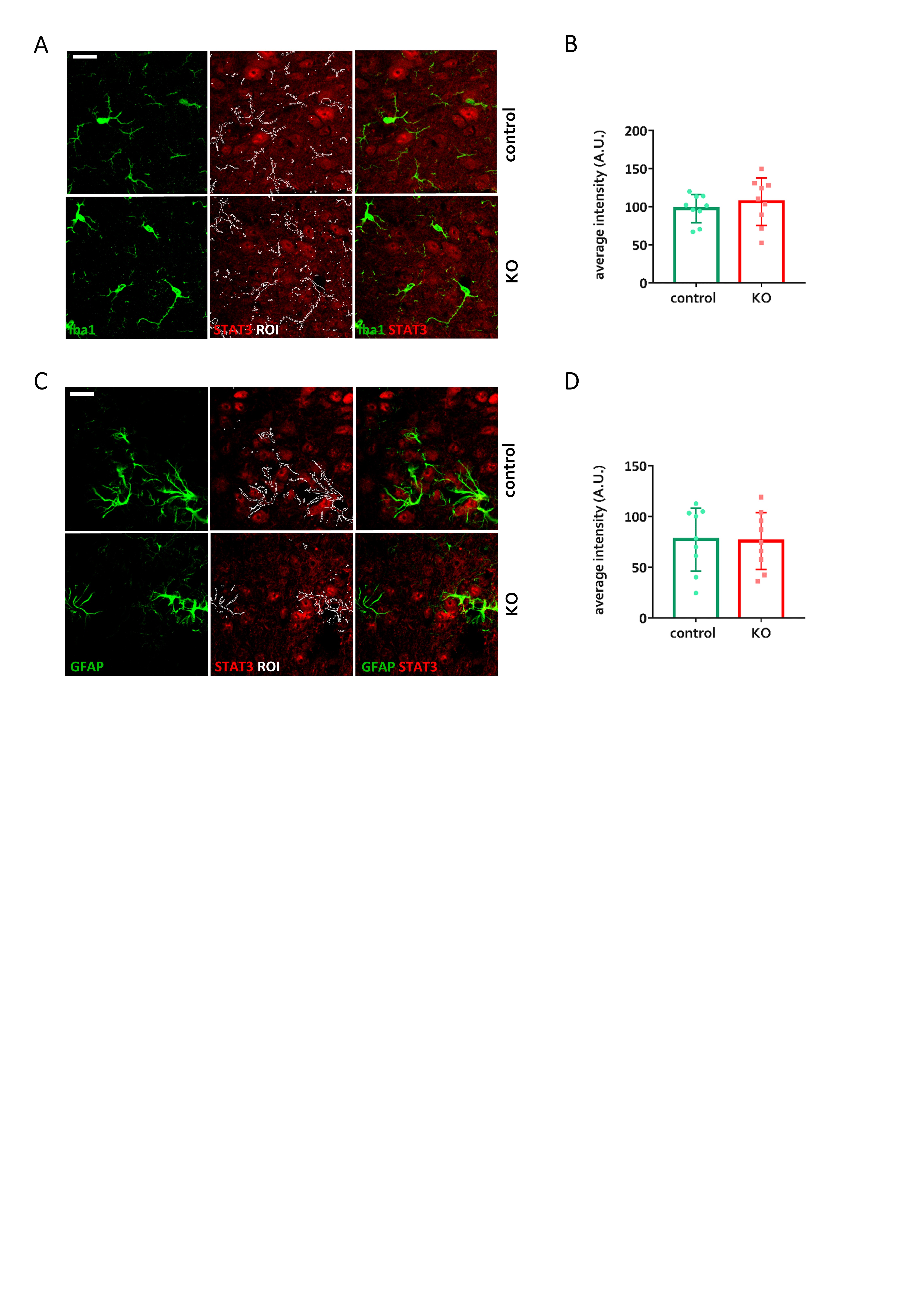

Supplement: Supplementary file 2 — Suppl Fig 1 [file 41380_2020_904_MOESM2_ESM.jpg]
