# Supplementary material for: STAT3 in the dorsal raphe gates behavioural reactivity and regulates gene networks associated with psychopathology
Source: Mol Psychiatry. 2020 Oct 12;26(7):2886–99. doi: 10.1038/s41380-020-00904-2 (PMC8505245; doi:10.1038/s41380-020-00904-2)
Supplement: Supplementary file 3 — Suppl Table 1 [file 41380_2020_904_MOESM3_ESM.pdf]

Supplementary Table 1

Model	Experiment	Figure	sample size / condition: control	sample size / condition: STAT3 deletion
genetic serotonergic STAT3 knockout	5HT/STAT3 immunofluorescence	1A-B	one 4x5 stitched image per section, 8 sections, 3 animals	one 4x5 stitched image per section, 10 sections, 3 animals
genetic serotonergic STAT3 knockout	Iba1/STAT3 immunofluorescence	1C-D	5 images per section, 9 sections, 3 animals	5 images per section, 9 sections, 3 animals
genetic serotonergic STAT3 knockout	GFAP/STAT3 immunofluorescence	1E-F	5 images per section, 9 sections, 3 animals	5 images per section, 9 sections, 3 animals
genetic serotonergic STAT3 knockout	DR electrophysiology	1H	6 animals	8 animals
genetic serotonergic STAT3 knockout	Open field	2A	26 animals	26 animals
genetic serotonergic STAT3 knockout	Rotarod	2B	24 animals	27 animals
genetic serotonergic STAT3 knockout	Sucrose preference test	2C	26 animals	24 animals
genetic serotonergic STAT3 knockout	Novelty-suppressed feeding: latency to feed	2D	23 animals	22 animals
genetic serotonergic STAT3 knockout	Novelty-suppressed feeding: body mass	2E	23 animals	22 animals
genetic serotonergic STAT3 knockout	Novelty-suppressed feeding: weight loss during food restriction	2F	23 animals	22 animals
genetic serotonergic STAT3 knockout	Novelty-suppressed feeding: food consumption post-test	2G	23 animals	22 animals
genetic serotonergic STAT3 knockout	Forced swim test	2H	23 animals	23 animals
genetic serotonergic STAT3 knockout	Light-dark box	2I	25 animals	24 animals
genetic serotonergic STAT3 knockout	Elevated plus maze	2J	25 animals	24 animals
genetic serotonergic STAT3 knockout	RNA-Seq	3A-E	4 animals	8 animals
genetic serotonergic STAT3 knockout	Amphetamine sensitisation	3F	8 animals	9 animals
genetic serotonergic STAT3 knockout	Conditioned place preference	3G	9 animals	8 animals

viral-mediated STAT3 knockdown	STAT3 immunofluorescence	4D	5 sections, 2 animals	6 sections, 2 animals
viral-mediated STAT3 knockdown	Transfection efficiency	4E	x	7 sections, 3 animals
viral-mediated STAT3 knockdown	Neural tropism	4E	x	5 sections, 2 animals
viral-mediated STAT3 knockdown	Open field	4F	9 animals	8 animals
viral-mediated STAT3 knockdown	Sucrose preference test	4G	9 animals	8 animals
viral-mediated STAT3 knockdown	Novelty-suppressed feeding: latency to feed	4H	8 animals	8 animals
viral-mediated STAT3 knockdown	Novelty-suppressed feeding: body mass	4I	8 animals	8 animals
viral-mediated STAT3 knockdown	Novelty-suppressed feeding: weight loss during food restriction	4J	8 animals	8 animals
viral-mediated STAT3 knockdown	Novelty-suppressed feeding: food consumption post-test	4K	8 animals	8 animals
viral-mediated STAT3 knockdown	Forced swim test	4L	9 animals	8 animals
viral-mediated STAT3 knockdown	Amphetamine sensitisation	4M	9 animals	8 animals
