# Supplementary material for: STAT3 in the dorsal raphe gates behavioural reactivity and regulates gene networks associated with psychopathology
Source: Mol Psychiatry. 2020 Oct 12;26(7):2886–99. doi: 10.1038/s41380-020-00904-2 (PMC8505245; doi:10.1038/s41380-020-00904-2)
Supplement: Supplementary file 4 — Suppl Table 2 [file 41380_2020_904_MOESM4_ESM.pdf]

Supplementary Table 2

Primary antibodies

Antigen	Species	Manufacturer	Catalog number	Experiment	Figure	Working concentration
STAT3	rabbit	Santa Cruz Biotechnology	sc-7179	5-HT/STAT3 immunofluorescence; STAT3 immunofluorescence in AAV-Cre and AAV-GFP cohorts	1A, 1B; 4C, 4D	1:100
STAT3	mouse	Abcam	ab119352	Iba1/STAT3 immunofluorescence; GFAP/STAT3 immunofluorescence	1C, 1D; 1E, 1F	1:100
5-HT	goat	Abcam	ab66047	5-HT/STAT3 immunofluorescence	1A, 1B	1:200
Iba1	rabbit	FUJIFILM Wako Pure Chemical Corporation	019-19741	Iba1/STAT3 immunofluorescence	1C, 1D	1:1000
GFAP	rabbit	Sigma-Aldrich	G9269	GFAP/STAT3 immunofluorescence	1D, 1F	1:500
NeuN	mouse	Chemicon/ Sigma-Aldrich	MAB377	NeuN immunofluorescence	4E	1:500

Primers for qRT-PCR

Target	Forward sequence (5' to 3')	Reverse sequence (5' to 3')
STAT3	GTTCTCGTCCACCACCAAGC	CCTTGCCAGCCATGTTTTCTTTG
β-actin	ATGGTGGGAATGGGTCAGAAG	TCTCCATGTCGTCCCAGTTG

Viral vectors

Group	Full name	Manufacturer	Serotype	Catalog number	Author
AAV-Cre	pAAV.CMV.HI.eGFP-Cre.WPRE.SV40	Addgene	AAV5	#105545-AAV5	James M. Wilson
AAV-GFP	pAAV.CMV.PI.EGFP.WPRE.bGH	Addgene	AAV5	#105530-AAV5	James M. Wilson
