# Supplementary material for: STAT3 in the dorsal raphe gates behavioural reactivity and regulates gene networks associated with psychopathology
Source: Mol Psychiatry. 2020 Oct 12;26(7):2886–99. doi: 10.1038/s41380-020-00904-2 (PMC8505245; doi:10.1038/s41380-020-00904-2)
Supplement: Supplementary file 6 — Suppl Table 3B [file 41380_2020_904_MOESM6_ESM.pdf]

Supplementary Table 3B

Enrichr analysis of DEGxSTAT3 (accessed 17.10.2019)

top 5 enriched processes (ranked by adjusted p value)

**GO: BP**	**adj. P**	**-log Benj**
monovalent inorganic cation transport (GO:0015672)	0.0003175	3.4982563
metal ion transport (GO:0030001)	0.0005063	3.2955921
nervous system development (GO:0007399)	0.0149000	1.8268137
potassium ion transport (GO:0006813)	0.0308600	1.5106041
regulation of neurotransmitter transport (GO:0051588)	0.0466300	1.3313346

**KEGG pathways**	**adj. P**	**-log Benj**
synaptic vesicle cycle	0.0209500	1.6788160
thyroid cancer	0.0853500	1.0687965
GnRH signaling pathway	0.0964300	1.0157878
calcium signaling pathway	0.0992500	1.0032695
Apelin signaling pathway	0.1048000	0.9796387
