# Supplementary material for: STAT3 in the dorsal raphe gates behavioural reactivity and regulates gene networks associated with psychopathology
Source: Mol Psychiatry. 2020 Oct 12;26(7):2886–99. doi: 10.1038/s41380-020-00904-2 (PMC8505245; doi:10.1038/s41380-020-00904-2)
Supplement: Supplementary file 8 — Suppl Table 3C [file 41380_2020_904_MOESM8_ESM.pdf]

Supplementary Table 3D

Enrichr analysis of DEGxSMI (accessed 17.10.2019)
top 5 enriched processes (ranked by adjusted p value)

GO: BP	adj. p	-log Benj
chemical synaptic transmission (GO:0007268)	0.0000009	6.0474497
anterograde trans-synaptic signaling (GO:0098916)	0.0072730	2.1382864
regulation of insulin secretion (GO:0050796)	0.0098670	2.0058149
neurotransmitter transport (GO:0006836)	0.0115600	1.9370422
nervous system development (GO:0044058)	0.0218800	1.6599527

KEGG pathways	adj. p	-log Benj
synaptic vesicle cycle	0.0000143	4.8443604
neuroactive ligand-receptor interaction	0.0004535	3.3434227
glutamatergic synapse	0.0008758	3.0575951
calcium signaling pathway	0.0009424	3.0257647
amphetamine addiction	0.0014770	2.8306195
